# Predicting Postoperative Complications in Lung Cancer Spinal Metastases: A Nomogram Based on Nutritional, Low Psoas Muscle Index, and Functional Status

**DOI:** 10.3390/curroncol33060336

**Published:** 2026-06-05

**Authors:** Xinyao Lv, Ruizhao Zhao, Yuyu Fan, Zijian Wang, Xiutong Fang, Junjie Qiao

**Affiliations:** Department of Orthopedics, Beijing Shijitan Hospital, Capital Medical University, Beijing 100038, China; lxy20000809@mail.ccmu.edu.cn (X.L.); ruizhao11@163.com (R.Z.); fanyy0507@163.com (Y.F.); 15652283881@163.com (Z.W.)

**Keywords:** lung cancer spinal metastases, postoperative complications, nomogram, low psoas muscle index, prognostic nutritional index

## Abstract

**Objective**: This study seeks to investigate the association between nutritional, muscular, and functional status and moderate-to-severe postoperative complications (Clavien–Dindo ≥ grade II) in patients with lung cancer spinal metastases and to construct an individualized risk prediction nomogram. **Methods**: A total of 162 patients with histologically confirmed lung cancer spinal metastases who underwent surgery were retrospectively enrolled. Preoperative clinical data were collected. Univariate and multivariate logistic regression analyses were used to identify independent risk factors for postoperative complications, with variable selection based on a combination of statistical significance and clinical judgment. A nomogram model was constructed and evaluated using receiver operating characteristic curve, calibration curve, and decision curve analysis. Internal validation was performed using the bootstrap method. For exploratory assessment of the model’s clinical stratification capability, patients were classified into low-, medium-, and high-risk groups based on tertiles of the predicted probability. **Results**: The incidence of Clavien–Dindo ≥ grade II complications during postoperative hospitalization or within 14 days was 57.4%. Multivariate analysis suggested that lower psoas muscle index (low PMI) (OR = 4.131, *p* = 0.034), lower body mass index (BMI) (continuous: OR = 0.539 per 1 kg/m^2^ increase, *p* = 0.001, indicating that lower BMI was associated with higher risk), lower prognostic nutritional index (PNI) (OR = 0.456, *p* < 0.001), and lower Karnofsky Performance Status (KPS) score (OR = 0.890, *p* = 0.009) were identified as potential independent factors associated with postoperative complications. The nomogram achieved an Area Under the Curve of 0.907, showed acceptable calibration (Hosmer–Lemeshow test, *p* = 0.735), and demonstrated a favorable net clinical benefit in the decision curve analysis. In the exploratory risk stratification analysis, complication rates in the low-, medium-, and high-risk groups were 28.8%, 63.6%, and 78.2%, respectively (*p* < 0.001). Patients with complications had significantly longer hospital stays (median 20 vs. 13 days). **Conclusions**: In this cohort, low PMI, low BMI, low PNI, and low KPS were identified as potential independent factors associated with short-term moderate-to-severe postoperative complications. The nomogram may preliminarily predict the risk and might serve as a quantitative reference for individualized perioperative management, but its clinical utility requires further confirmation in external validation. The exploratory risk stratification suggests that the model has preliminary potential for clinical discrimination.

## Simple Summary

Lung cancer that spreads to the spine often requires surgery, but these patients have a high risk of moderate-to-severe complications after the operation. Currently, doctors lack a simple tool to predict this risk before surgery. In this study, we analyzed 162 patients and found that four factors—low psoas muscle index (a measure of muscle mass), low body weight, poor nutrition–immune status, and poor daily function—were independent predictors of complications. We then combined these factors into a user-friendly chart (nomogram) that estimates each patient’s risk before surgery. This tool can help doctors identify high-risk patients early, plan better preoperative support, and reduce complications. Future studies should test this tool in other hospitals to confirm its usefulness.

## 1. Introduction

Lung cancer is not only the malignancy with the highest incidence and mortality worldwide but also one of the tumor types most prone to bone metastasis. Among these, the spine is the most common metastatic site, accounting for 30–50% of all spinal metastases cases, making this population a key target for risk stratification [1,2,3]. Spinal metastases from lung cancer frequently lead to intractable back pain, pathological fractures, spinal cord compression, and neurological deficits, severely impairing quality of life and increasing healthcare burden [4,5]. For eligible patients, surgery can relieve spinal cord compression, restore spinal stability, improve neurological function, and alleviate pain [6,7,8].

However, most patients with lung cancer spinal metastases present with advanced disease, accompanied by systemic wasting, malnutrition, and immunosuppression. Compared to spinal metastases from other primary tumors (e.g., breast, prostate, or renal cancer), lung cancer metastases are associated with worse prognosis, shorter expected survival, and higher perioperative complication risk [9]. This is not only due to the aggressive biological behavior of lung cancer and its frequent occult dissemination but also closely related to compromised host nutritional and immune status [10]. The surgical trauma is substantial, and the postoperative complication rate reaches 30–40%, which prolongs hospital stay, increases medical costs, delays subsequent antitumor therapy, and may even compromise long-term survival [11,12,13]. Therefore, accurate preoperative identification of high-risk patients is an urgent clinical need. Furthermore, lung cancer is highly heterogeneous. Different histological subtypes (e.g., adenocarcinoma, squamous cell carcinoma, and small cell carcinoma) exhibit variable responses to systemic therapy and disease control, and this heterogeneity may further influence postoperative recovery.

In recent years, the role of nutritional–immune status and low psoas muscle index (PMI) in the prognostic assessment of oncological surgery has received increasing attention [10,14]. Although the value of the prognostic nutritional index (PNI) and low PMI in tumor prognosis has been widely recognized, most existing studies focus on single indicators, lacking systematic multidimensional risk prediction tools [10,15]. Clinical assessment of postoperative complications in patients with lung cancer spinal metastases still relies mainly on physician experience, lacking standardized quantitative models and resulting in delayed identification of high-risk patients and difficulty in achieving early intervention. In the field of spinal surgery, some studies have attempted to construct prediction models for postoperative complications [16], but models specifically targeting the special population of lung cancer spinal metastases remain scarce. Therefore, through a retrospective analysis, this study aims to: (1) preliminarily investigate the association of PNI, low PMI, and other preoperative clinical factors with postoperative Clavien–Dindo ≥ grade II complications in patients with lung cancer spinal metastases; (2) identify potential independent risk factors; (3) attempt to construct a nomogram based on preoperatively available variables as an exploratory tool for individualized risk prediction. This tool may provide a reference for clinicians in the early identification of high-risk patients and offer a preliminary direction for future prospective validation and intervention studies. Additionally, we descriptively evaluated the impact of complications on postoperative hospital stay to explore its potential clinical and economic burden.

## 2. Materials and Methods

### 2.1. Patient Population

This was a single-center, retrospective cohort study. Clinical records of patients who presented to the Department of Spine Surgery, Beijing Shijitan Hospital, Capital Medical University, from January 2019 to October 2024 for lung cancer spinal metastases were analyzed. Patients who strictly met the inclusion/exclusion criteria and had complete perioperative data were included in this study. Finally, 162 patients aged 18 years or older with pathologically confirmed lung cancer spinal metastases were enrolled.

Patient medical records were obtained through the hospital medical records management system. Data collected included age, gender, body mass index (BMI), hypertension (blood pressure ≥ 140/90 mmHg), diabetes mellitus (DM) (fasting blood glucose ≥ 6.1 mmol/L), number of extraspinal metastases, site of spinal metastases, preoperative Day 1 albumin (ALB) level, preoperative hemoglobin (HB) level, preoperative lymphocyte count, and preoperative Day 1 Karnofsky Performance Status (KPS) score. In addition, the following surgery-related variables were collected to adjust for the potential confounding effect of surgical invasiveness on outcomes: operative time (hours), estimated intraoperative blood loss (mL), number of instrumented levels, and use of robotic assistance (Tianji Orthopedic Robotic System, TINAVI Medical Technologies, Beijing, China). Prognostic scores used for surgical decision-making—the revised Tokuhashi score and the Tomita score—were also recorded. Pathological reports were reviewed to determine histological subtype (adenocarcinoma, squamous cell carcinoma, and small cell lung cancer). Postoperative in-hospital complications were independently recorded by two clinicians and classified according to the Clavien–Dindo classification system. Complications were defined as grade ≥ II events occurring during hospitalization or within 14 days postoperatively. Inter-observer consistency was assessed using the Kappa test, yielding a Kappa value of 0.77 (95% CI: 0.69–0.85). Informed consent was obtained from all patients for the clinical data involved in this study. All methods were performed in accordance with the relevant guidelines and regulations of the Declaration of Helsinki. This study was approved by the Medical Ethics Committee of Beijing Shijitan Hospital affiliated with Capital Medical University (Ethics Approval Number: IIT2024-127-002).

### 2.2. Inclusion and Exclusion Criteria

Surgical indication criteria: At our institution, surgical decision-making for spinal metastases from lung cancer was guided by the revised Tokuhashi scoring system and the Tomita score, both of which were routinely recorded in the clinical workup. The specific criteria for considering surgery were: (1) a revised Tokuhashi score ≥ 8 (indicating an expected survival of ≥6 months); (2) a Tomita score of 2–7 (indicating that surgery could offer survival benefit or palliative improvement); and (3) the presence of one or more of the incidence of progressive neurological deficit due to epidural spinal cord compression or intractable pain refractory to radiotherapy.

All 162 included patients met at least the Tokuhashi/Tomita criteria for surgical candidacy. Accordingly, the nomogram developed herein applies exclusively to patients who have already been selected for surgery based on these prognostic scores and should not be extrapolated to unselected populations or to patients with extremely poor expected survival.

Inclusion criteria were as follows: ① patients with metastatic tumors of the cervical, thoracic, and lumbar spine confirmed by imaging (e.g., X-ray, CT, and MRI) and histopathological examination showing lung cancer (with or without concurrent extraspinal metastases); ② first-time surgical treatment for spinal metastases from lung cancer; ③ complete preoperative and postoperative routine blood test records (including at least HB, ALB, and lymphocyte counts) and detailed anesthesia records.

Exclusion criteria were as follows: ① combined severe hematological diseases, including but not limited to known coagulation disorders (e.g., hemophilia), platelet count < 100 × 10^9^/L, or diseases severely affecting coagulation function (e.g., liver cirrhosis Child–Pugh grade B/C, etc.); ② severe insufficiency of important organ functions, such as cardiac function NYHA grade III–IV; liver function (ALT/AST > 2 times the upper limit of normal); and renal function (creatinine clearance < 30 mL/min); ③ use of therapeutic doses of anticoagulant drugs (e.g., warfarin, rivaroxaban) or antiplatelet agents (e.g., aspirin, clopidogrel) within 1 week preoperatively; ④ preoperative treatment with therapeutic doses of low-molecular-weight heparin injection for deep vein thrombosis or other diseases; ⑤ history of spinal surgery at the same site; ⑥ receipt of radiotherapy or embolization targeting the spinal metastasis or systemic chemotherapy within 1 month preoperatively.

### 2.3. Surgical Technique

All surgeries were performed by the same spinal surgery team with over 20 years of experience. Surgical plans were individualized based on the location, extent, and degree of vertebral destruction of the spinal metastases, with the core strategy being safe tumor debulking combined with posterior pedicle screw-rod system stabilization, supplemented by anterior column reconstruction (vertebroplasty or balloon kyphoplasty) based on vertebral body defects. All surgeries were performed via a posterior midline approach. The main steps included pedicle screw placement (robot-assisted using the Tianji Orthopedic Robotic System or conventional freehand placement depending on intraoperative conditions), anterior column augmentation, and tumor debulking. Intraoperative attention was paid to protecting adjacent neurovascular structures, and routine postoperative drainage was applied.

The extent of debulking was adjusted individually based on intraoperative frozen section pathology and tumor involvement, without enforcing a fixed resection margin, with the primary goals being adequate spinal cord decompression and restoration of spinal stability. To adjust for the potential confounding effect of surgical invasiveness on postoperative complications, the following surgery-related variables were routinely recorded and incorporated into the statistical analysis: operative time (hours), estimated intraoperative blood loss (mL), number of instrumented levels and use of robotic assistance.

### 2.4. Preoperative PNI

The PNI was initially proposed by Buzby and subsequently refined by Onodera and colleagues in Japan. As a nutritional assessment tool and surgical risk predictor, it was originally designed to evaluate perioperative status and predict surgical risk in patients with gastrointestinal tumors [17,18,19].

The PNI is a concise index comprising two parameters: serum ALB and total lymphocyte count (TLC). ALB, synthesized by the liver, is a key plasma component that maintains colloid osmotic pressure. Long-term inadequate protein intake leads to decreased ALB levels, making it an indicator of chronic protein malnutrition and reflecting overall nutritional status. However, some recent studies have shown that PNI can also assess the aggressiveness of malignant tumors [20,21]. The formula for calculating PNI is

(1) $$PNI=ALB\;(g/L)+5\times TLC\;(10^{9}/L)$$

In lung cancer-related research, the PNI is widely used to assess patients’ nutritional–immune status and its association with clinical prognosis [22]. Multiple studies have confirmed that lower PNI levels are significantly associated with increased risk of postoperative complications and decreased long-term survival rates [23,24,25]. In this study, the PNI was treated as a continuous variable to investigate the impact of preoperative nutritional–immune status on postoperative outcomes in patients with lung cancer spinal metastases, allowing for a more precise analysis of its relationship with prognosis.

### 2.5. Psoas Muscle Area

The cross-sectional area of the psoas muscle at the L3 vertebral body level was measured on T2-weighted axial MRI images acquired using a 3.0T MRI scanner (MAGNETOM Skyra, Siemens Healthcare, Erlangen, Germany). Two spine surgeons blinded to clinical outcomes independently performed the measurements, and the average value was used for analysis. The inter-observer reliability was assessed using the intraclass correlation coefficient (ICC), which was 0.90 (95% CI: 0.86–0.93).

**Figure 1:**
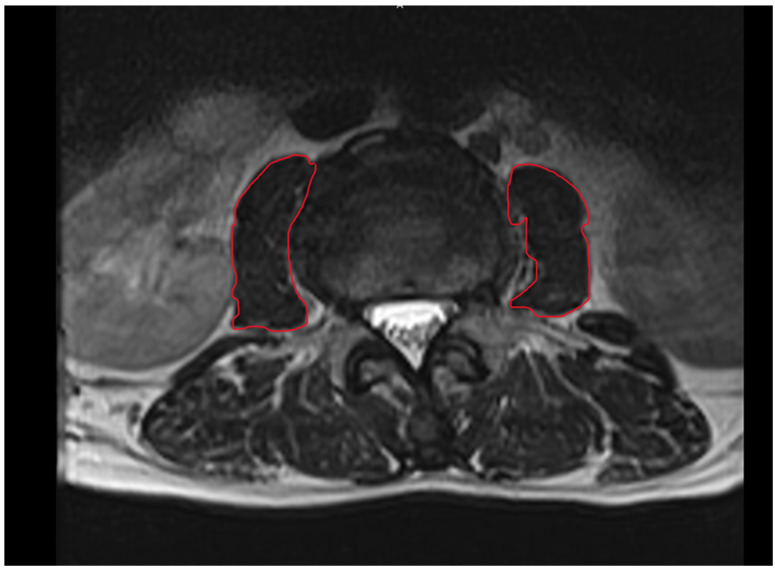
Measurement of the psoas muscle at the L3 vertebral body level.

The psoas muscle index (PMI) was calculated as psoas muscle area (cm^2^)/height^2^ (m^2^). Based solely on muscle quantity, patients with a PMI below the Asian diagnostic thresholds (male < 6.36 cm^2^/m^2^, female < 3.92 cm^2^/m^2^) were classified as having low PMI [26]. It is important to note that this definition assesses only the muscle mass component and does not capture muscle strength or physical performance and thus is not equivalent to a comprehensive diagnosis of sarcopenia. In addition to this binary variable, the PMI was also analyzed as a continuous variable in the multivariable regression to evaluate the linear relationship between each unit change in psoas muscle index and the risk of postoperative complications (Figure 1).

### 2.6. Statistical Analysis

Statistical analyses were performed using SPSS 27.0 (IBM Corp., Armonk, NY, USA) and RStudio 4.5.2 (Posit Software, PBC, Boston, MA, USA). Normality of continuous variables was tested by the Shapiro–Wilk test; normally distributed variables were expressed as mean ± SD and non-normally distributed variables as median (IQR). Univariate binary logistic regression was used to screen potential factors for postoperative Clavien–Dindo ≥ grade II complications.

To adjust for the impact of surgical invasiveness on postoperative complications, we included operative time, estimated intraoperative blood loss, and number of instrumented levels as the core surgical covariates in the multivariable regression models. These three variables collectively capture the major dimensions of surgical invasiveness, namely, procedural duration, intraoperative tissue trauma, and extent of spinal fixation. In contrast, robot-assisted navigation was not included because its primary effect is on pedicle screw placement accuracy rather than on overall surgical invasiveness. Regarding the Tomita classification of tumor extent and histological subtype, both had highly uneven distributions in our cohort, and including them would lead to unstable model estimates; therefore, they were not included in the regression model.

Variable selection for multivariable logistic regression followed a predefined strategy: **①** variables significant in univariate analysis (low PMI, BMI, PNI, KPS, surgical duration, and number of instrumented levels) were included; **②** continuous PMI was forced into the model to test whether it had predictive value independent of the categorical low PMI; **③** based on clinical judgment, surgical complexity (intraoperative blood loss per 100 mL) and tumor prognosis (preoperative Tokuhashi score) were forced into the model to control for confounding.

Collinearity was assessed using the variance inflation factor (VIF); all VIF values were <5, indicating no significant multicollinearity. The above surgery-related variables used for confounding control (operative time, number of instrumented levels, intraoperative blood loss, etc.) were retained in the multivariable model regardless of their statistical significance. These variables were used only for confounder adjustment and were not incorporated into the final nomogram prediction tool.

The final nomogram was constructed using a simplified logistic regression model that included only the four predictors (low PMI, BMI, PNI, and KPS). The multivariable analysis that additionally adjusted for surgical covariates (operative time, number of instrumented levels, intraoperative blood loss, and Tokuhashi score) was performed solely to confirm the robustness of these independent predictors after controlling for surgical complexity; its results were not used to derive the nomogram coefficients (see Section 3 for the full multivariable analysis).

A total of 93 outcome events occurred, and the final model included four independent predictors (low PMI, BMI, PNI, and KPS), yielding an event-to-variable ratio of approximately 23:1, exceeding the recommended minimum threshold of 10:1 and suggesting a low risk of overfitting. No missing data were present for the primary analysis variables (BMI, PNI, KPS, PMI, etc.); missing values occurred only in non-core variables (e.g., specific chemotherapy regimens, socioeconomic status), which were not included in the analysis, and thus no imputation was applied.

The prediction model was constructed based on multivariable logistic regression results and internally validated using bootstrap resampling (1000 replicates). Discrimination was assessed by the AUC; calibration by the Hosmer–Lemeshow test, calibration curve, and Brier score; and clinical utility by decision curve analysis (DCA). A nomogram was plotted for model visualization. Patients were divided into low-, medium-, and high-risk groups based on tertiles of the predicted probability, and comparisons were made using the chi-square test with Bonferroni correction. The significance level α was set at 0.05.

## 3. Results

**Figure 2:**
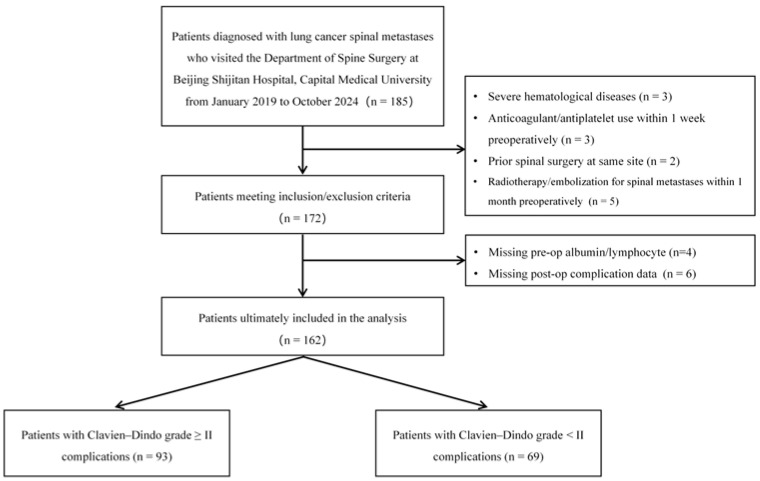
Flowchart of the patient enrollment process.

The patient screening process is shown in Figure 2. After rigorous inclusion and exclusion criteria, a total of 162 patients with lung cancer spinal metastases were included in the analysis.

A total of 162 patients were retrospectively reviewed. Among them, 108 were male and 54 were female, with a mean age of 66.65 ± 9.85 years (range 18–86 years). Table 1 presents the demographic and clinical baseline characteristics. The mean BMI was 22.27 ± 3.76 kg/m^2^, mean preoperative hemoglobin was 125.54 ± 14.28 g/L, mean PNI was 44.53 ± 4.33, and mean preoperative KPS score was 55.19 ± 10.70. The mean PMI was 3.12 ± 0.87 cm^2^/m^2^. The median surgical duration was 2.76 ±0.83 h, the median number of instrumented levels was 3.66 ±1.27, and the median postoperative hospital stay was 16 (7,25) days. The mean Tokuhashi score was 10.91 ± 1.90, and the mean Tomita score was 4.62 ± 1.66. Postoperative grade ≥ II complications occurred in 93 patients (57.4%), and low PMI was present in 118 patients (72.8%). Regarding histological subtype distribution, there were 123 patients (75.9%) with adenocarcinoma, 21 (13.0%) with squamous cell carcinoma, and 18 (11.1%) with small cell lung cancer.

Taking the occurrence of postoperative Clavien–Dindo ≥ grade II complications as the dependent variable, univariate binary logistic regression analysis was performed on patient clinical factors, with results shown in Table 2. Univariate analysis showed that low PMI (OR = 17.774, 95% CI: 6.849–46.126, *p* < 0.001), BMI (OR = 0.452, 95% CI: 0.355–0.577, *p* < 0.001), preoperative PNI (OR = 0.407, 95% CI: 0.309–0.536, *p* < 0.001), preoperative KPS (OR = 0.862, 95% CI: 0.824–0.902, *p* < 0.001), number of instrumented levels (OR = 1.248, 95% CI: 1.076–1.448, *p* = 0.003), and surgical duration (OR = 1.302, 95% CI: 1.082–1.567, *p* = 0.005) were significantly associated with postoperative complications. Preoperative HB level (*p* = 0.065) and history of DM/hypertension (*p* > 0.05) did not reach statistical significance in the univariate analysis.

Before constructing the multivariate regression model, collinearity diagnostics were performed on the candidate variables, with results shown in Table 3. The tolerance of each variable was >0.2, and the VIF was <5, indicating no significant multicollinearity among the variables, which could be simultaneously included in the regression model for analysis.

Variables that were statistically significant in the univariate analysis (low PMI, BMI, PNI, KPS, number of instrumented levels, and surgical duration) as well as clinically important variables (continuous PMI, intraoperative blood loss, and Tokuhashi score) were included together in the multivariate binary logistic regression model. The analysis results are shown in Table 4. After adjusting for confounding factors, low PMI (OR = 4.131, 95% CI: 1.158–23.926, *p* = 0.034), BMI (OR = 0.539, 95% CI: 0.372–0.783, *p* = 0.001), preoperative PNI (OR = 0.456, 95% CI: 0.326–0.638, *p* < 0.001), and preoperative KPS (OR = 0.890, 95% CI: 0.816–0.971, *p* = 0.009) remained statistically significant, indicating that the above indicators are independent factors influencing the occurrence of postoperative grade ≥ II complications in patients with lung cancer spinal metastases. However, surgical duration, continuous PMI, Tokuhashi score, number of instrumented levels, and intraoperative blood loss were not statistically significant in the multivariable analysis (all *p* > 0.05) and thus were not included in the final nomogram.

**Table 4:** Multivariate analysis of binary logistic regression for postoperative complications.

Influencing Factors	β Value	Wald	*p* Value	OR Value	95% CI
BMI	−0.617	10.549	0.001	0.539	0.372–0.783
PMI	0.282	0.432	0.511	1.325	0.572–3.070
Low PMI	1.964	4.485	0.034	4.131	1.158–23.926
Preoperative PNI	−0.786	21.027	<0.001	0.456	0.326–0.638
Preoperative KPS	−0.116	6.833	0.009	0.890	0.816–0.971
Surgical duration	0.839	3.417	0.065	2.313	0.951–5.628
Tokuhashi score	−0.085	0.151	0.698	0.919	0.599–1.409
Instrumented levels	−0.604	2.460	0.117	0.546	0.257–1.163
Intraoperative blood loss (per 100 mL)	−0.066	0.927	0.336	0.936	0.819–1.071

Using the median PNI as the cutoff, patients were divided into a low PNI group (*n* = 81) and a high PNI group (*n* = 81). The incidence of postoperative complications in the low PNI group was significantly higher than that in the high PNI group (*p* < 0.001). Similarly, the complication rate in patients with low PMI was significantly higher than that in patients without low PMI (*p* < 0.001). Detailed stratification data are shown in Table 5.

Among the 93 patients with Clavien–Dindo ≥ grade II complications, the distribution of specific complication categories is shown in Table 6. Hypoalbuminemia was the most common (59 patients, 63.4%), followed by infectious events (41 patients, 44.1%), electrolyte disturbances (34 patients, 36.6%), and anemia (27 patients, 29.0%). Thromboembolic events occurred in 16 patients (17.2%), and other complications (pressure ulcer, arrhythmia, respiratory failure, etc.) were noted in 22 patients (23.7%).

Among the 162 patients, 123 (75.9%) had adenocarcinoma, 21 (13.0%) had squamous cell carcinoma, and 18 (11.1%) had small cell lung cancer. The incidence of postoperative Clavien–Dindo ≥ grade II complications was 56.1% (69/123) for adenocarcinoma, 57.1% (12/21) for squamous cell carcinoma, and 66.7% (12/18) for small cell lung cancer (Table 7).

**Figure 3:**
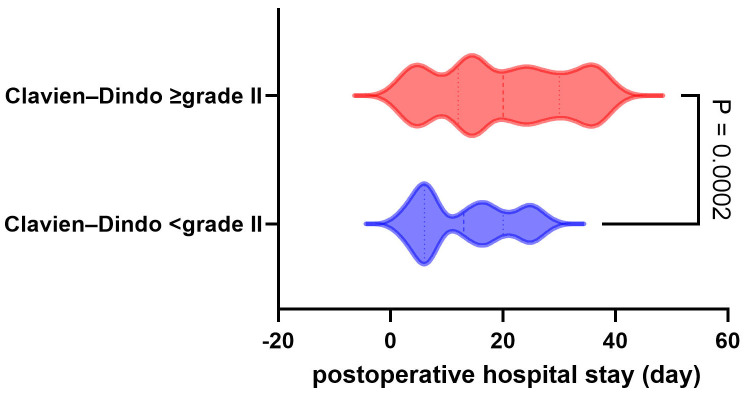
Comparison of postoperative hospital stay between patients with Clavien–Dindo < grade II and ≥ grade II.

In the health–economic and clinical burden analysis, patients with postoperative Clavien–Dindo ≥ grade II complications (*n* = 93) had a significantly longer postoperative hospital stay (median [IQR]: 20.0 [12.0–30.0] days vs. 13.0 [6.0–20.0] days, *p* < 0.001) (Figure 3).

To further explore the association between different risk factors and specific complication types, we descriptively stratified the incidence of major complications by preoperative PNI (median cutoff 44.125) and low PMI status, as shown in Table 8. In the low PNI group, the incidence of infectious events was 44.4%, substantially higher than the 6.2% in the high-PNI group; hypoalbuminemia occurred in 67.9% of the low-PNI group versus only 4.9% in the high-PNI group. Similarly, in the low PMI group, the incidence of infectious events was 33.1%, compared with 4.5% in the group without low PMI; hypoalbuminemia occurred in 47.5% of the low PMI group versus 6.8% in the group without low PMI. Thromboembolic events showed a similar pattern in the low PNI (17.2% vs. 2.5%) and low PMI (12.7% vs. 2.3%) subgroups. It should be emphasized that these stratified analyses are purely descriptive, without multivariable adjustment or hypothesis testing, and the findings should be considered exploratory.

Based on the results of the multivariate logistic regression analysis, a risk prediction model for postoperative Clavien–Dindo ≥ grade II complications was constructed. The regression equation is as follows:

(2) Logit(p) = 55.532 + 1.964 × low PMI (1 = present, 0 = absent) − 0.617 × BMI − 0.786 × PNI − 0.116 × KPS

**Figure 4:**
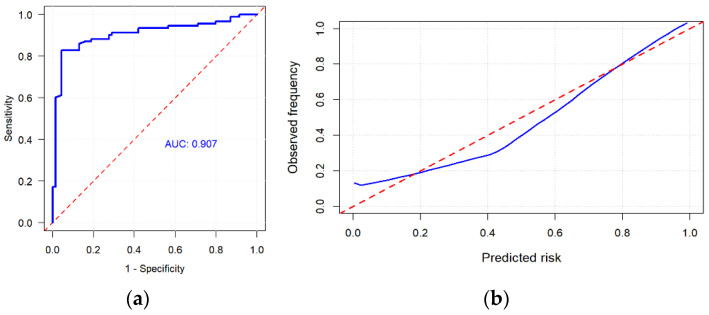
(**a**) ROC curve of the prediction model; (**b**) calibration curve of the prediction model.

The bootstrap method with 1000 resamples was used for internal validation of the model development data. The AUC value was 0.907 (95% CI, 0.858–0.957), and the Brier score was 0.114 (95% CI, 0.073–0.155). The calibration curve showed that the predicted probability of postoperative Clavien–Dindo grade ≥ II complications was highly consistent with the actual probability (Figure 4), indicating good predictive accuracy. The H-L test yielded a χ^2^ value of 5.212, *p* = 0.735, indicating no statistically significant difference between predicted risk and actual risk.

**Figure 5:**
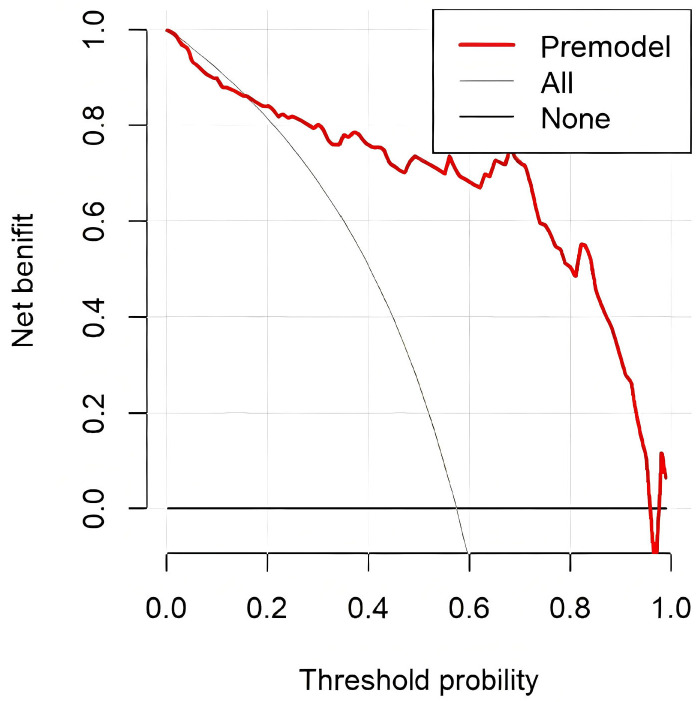
DCA of the prediction model.

Furthermore, the clinical validity of the model was assessed using DCA, which showed that within the probability threshold range of approximately 0.15 to 0.95, clinical decisions made using the model were more beneficial than the “no intervention” or “intervention for all” options (Figure 5).

**Figure 6:**
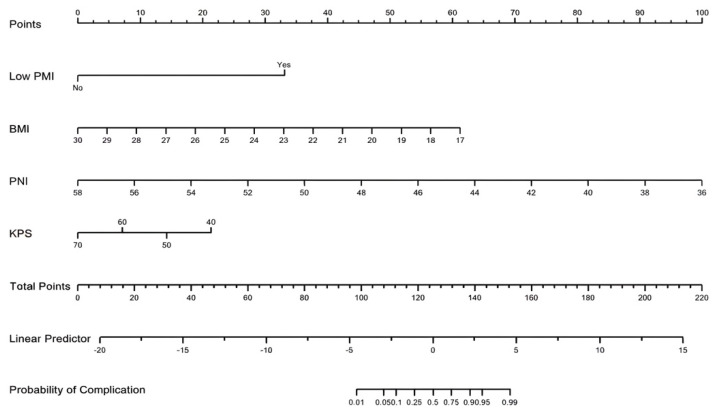
Nomogram for predicting the risk of postoperative Clavien–Dindo ≥ grade II complications in patients with lung cancer spinal metastases.

This study used RStudio (version 4.5.2) to construct a nomogram for predicting the risk of postoperative Clavien–Dindo grade ≥ II complications in patients with lung cancer spinal metastases and visualized the model. This nomogram was based on four independent influencing factors identified by multivariate logistic regression analysis: low PMI, BMI, PNI, and preoperative KPS. In the nomogram, each predictor corresponds to a point value. The total score is obtained by summing the points of each variable, which is then mapped to the predicted probability of a patient experiencing postoperative grade ≥ II complications. As an exploratory analytical tool, this nomogram can preliminarily provide clinicians with a quantitative reference for risk probability and, after external validation, may be used to guide individualized perioperative management (Figure 6).

To exploratorily assess the clinical stratification ability of the model, the 162 patients were divided into three groups based on tertiles of the predicted probability, with results shown in Table 9. The incidence of postoperative Clavien–Dindo ≥ grade II complications in the low-, medium-, and high-risk groups was 28.8%, 63.6%, and 78.2%, respectively, showing an increasing trend. This stratification is based on the distribution of predicted probabilities in this dataset and is an exploratory analysis; its cutoff values need further confirmation in external validation.

## 4. Discussion

Currently, commonly used prediction models in the field of spinal metastases (such as Tokuhashi score, Tomita score, the machine learning-based SORG algorithm, etc.) are mainly used for surgical decision-making and survival prognosis assessment. They have not taken short-term postoperative complications as a core prediction endpoint, and these models mostly rely on tumor characteristics (e.g., primary tumor type and visceral metastases) and general performance status, without incorporating objective nutritional and low PMI assessments [27,28,29,30,31]. Our nomogram integrates nutritional status (PNI), muscle mass (low PMI), body habitus (BMI), and functional status (KPS), providing a multidimensional assessment of patients’ physiological reserve.

Through a retrospective analysis of 162 patients with lung cancer spinal metastases, this study found that the incidence of postoperative Clavien–Dindo ≥ grade II complications was 57.4%, and the prevalence of low PMI was 72.8%. This relatively high complication rate may be related to the fact that most of the included patients had advanced lung cancer spinal metastases, with substantial surgical trauma and poorer systemic nutritional status, leading to higher postoperative risk than in the general spinal tumor population. Multivariate logistic regression analysis showed that low PMI, BMI, preoperative PNI, and preoperative KPS were independent factors influencing the occurrence of postoperative grade ≥ II complications. The nomogram prediction model constructed based on the above four indicators demonstrated good predictive performance in internal validation: after 1000 bootstrap resamples, the model achieved an AUC of 0.907 (95% CI: 0.858–0.957), indicating excellent discrimination ability; the calibration curve showed high consistency between predicted and observed probabilities, with an H-L test *p* = 0.735, suggesting good model calibration; the DCA indicated that using this model for clinical decision-making within the probability threshold range of 0.15 to 0.95 provided better net benefit than “intervention for all” or “no intervention” strategies, showing certain clinical utility potential. These results suggest that this nomogram may help preliminarily identify patients at high risk of postoperative complications and could serve as a potential reference tool for clinical decision-making.

Our results showed that both low PMI (muscle mass reduction defined by Asian criteria) and low BMI were independent risk/protective factors for moderate-to-severe postoperative complications (low PMI: OR = 4.131, *p* = 0.034; BMI: OR = 0.539, *p* = 0.001). However, the interpretation of these two indicators requires caution and should be integrated. First, low PMI in this study was defined solely based on muscle quantity (cross-sectional psoas area at L3 on MRI) without incorporating muscle strength or physical performance; therefore, it represents low muscle mass rather than the full clinical syndrome of sarcopenia. Notably, when PMI was analyzed as a continuous variable, it was not significantly associated with complications, suggesting a threshold effect: only severe muscle depletion below the sex-specific cutoff significantly increases risk. A low PMI not only reflects muscle wasting but is also often accompanied by a systemic chronic low-grade inflammatory state, which may exacerbate the stress response after surgical trauma [32]. Previous studies have confirmed that a low PMI is an independent predictor of postoperative complications and poor prognosis in lung cancer surgery [10,33,34]. Second, the BMI is a composite measure that cannot distinguish fat mass from muscle mass. Hence, the “protective effect” of a higher BMI observed in this study should not be simplistically equated with “better nutritional or physiological reserve.” A more plausible explanation is that the BMI reflects overall metabolic reserve (including subcutaneous fat and some lean mass), whereas a low PMI indicates reduced core muscle mass. The fact that both the BMI and a low PMI remained independent predictors in the same multivariable model indicates that they capture different dimensions of patients’ physiological vulnerability: a low PMI indicates muscle depletion, while a low BMI indicates insufficient overall body mass. When both coexist (i.e., a low BMI combined with a low PMI), the patient’s physiological reserve is likely severely exhausted, leading to a superimposed or even synergistic increase in complication risk.

In patients with lung cancer, factors such as tumor consumption, systemic inflammatory response, and decreased appetite often lead to malnutrition and immune suppression, manifested as a decreased PNI [35]. The prognostic value of the PNI stems precisely from its dual reflection of nutrition and immunity: hypoalbuminemia not only indicates visceral protein depletion but is also closely related to inflammatory states mediated by pro-inflammatory cytokines such as IL-6 and TNF-α, which can downregulate ALB synthesis [36]; lymphocytopenia implies impaired cellular immunity, increasing the risk of postoperative infection [37]. It should be noted that the PNI is not a pure nutritional indicator, and its levels may also be influenced by factors such as tumor-related inflammatory response, liver synthetic function, and fluid status. Therefore, its association with complications may reflect a broader imbalance in host status [38,39]. A low PNI often indicates decreased protein synthesis capacity and weakened immune function, increasing the risk of complications such as postoperative infection and poor wound healing. In the field of spinal surgery, the PNI has been confirmed as an effective indicator for predicting postoperative complications and survival in patients with spinal metastases [23,24,25]. This study further suggests that preoperative PNI may be an independent protective factor against moderate-to-severe postoperative complications in patients with lung cancer spinal metastases, suggesting that clinical attention should be paid to the assessment of preoperative nutritional–immune status.

Preoperative KPS also showed an independent protective effect in this study. KPS is an important tool for assessing the functional status and quality of life of cancer patients, reflecting their overall health and daily activity ability [40]. Patients with higher preoperative KPS generally have better physiological reserve and functional status, can better tolerate surgical trauma, and have stronger postoperative recovery ability [41]. For patients with a lower KPS, this suggests potential frailty or functional impairment, leading to a higher risk of postoperative complications. Therefore, preoperative assessment of functional status should also be emphasized.

The four indicators identified in this study are not mutually exclusive but collectively characterize patients’ perioperative physiological reserve from different dimensions: the PNI reflects the nutrition–immunity axis, a low PMI reflects skeletal muscle reserve, the BMI reflects overall body habitus and metabolic reserve, and the KPS reflects functional status. Modeling with all four together is superior to using any single indicator, suggesting that the risk of postoperative complications in patients with lung cancer spinal metastases is essentially closely related to multidimensional vulnerability.

In the univariate analysis of this study, operative time, number of instrumented levels, and intraoperative blood loss were significantly associated with postoperative complications. Therefore, these variables were forced into the multivariable logistic regression model as covariates for surgical invasiveness to adjust for their potential confounding effect on the predictive value of nutritional–muscular parameters. However, in the multivariable analysis, none of the above surgery-related variables reached statistical significance. Given that a nomogram, as a clinical prediction tool, should be as parsimonious as possible and based on easily obtainable preoperative variables, we ultimately retained only the four variables (low PMI, BMI, PNI, and KPS) that remained consistently significant in the multivariable analysis. It should be emphasized that although the surgery-related variables did not enter the final model, their role in confounder adjustment is indispensable; the conclusion that “nutritional-muscular parameters are independent predictors” was reached precisely after adjusting for surgical complexity.

Among moderate-to-severe postoperative complications, hypoalbuminemia (63.4%) and infectious events (44.1%) were the most prominent. The high prevalence of hypoalbuminemia indicates severe preoperative visceral protein depletion, a major risk factor for postoperative infection and poor wound healing [42,43]. Notably, the low-PNI group had a much higher incidence of infectious events (44.4% vs. 6.2%) and hypoalbuminemia (67.9% vs. 4.9%) compared with the high-PNI group, providing intuitive evidence for the predictive value of the PNI as a composite nutrition–immunity index. Furthermore, patients with a low PMI had higher incidences of infectious events (33.1% vs. 4.5%) and thromboembolic events (12.7% vs. 2.3%) than those without a low PMI, suggesting that muscle depletion may increase complication risk via chronic inflammation and hypercoagulability. These descriptive observations offer preliminary clues for differential mechanisms linking risk factors to specific complication types but require validation in larger cohorts.

This study further quantified the clinical and health–economic burden associated with postoperative complications. We found that patients with Clavien–Dindo ≥ grade II complications had a median postoperative hospital stay prolonged by 7 days (20 days vs. 13 days). Prolonged hospital stay not only increases healthcare resource utilization and direct medical costs but may also delay the initiation of subsequent antitumor therapy (e.g., radiotherapy, targeted therapy, or immunotherapy), thereby affecting patients’ long-term prognosis [44,45].

This study performed an exploratory risk stratification analysis based on tertiles of the model’s predicted probability. It should be emphasized that this stratification method originated from the distribution characteristics of this dataset, and its cutoff values (0.478 and 0.708) are not cross-sample stable and should not be directly used as clinical decision thresholds. Under this exploratory stratification framework, high-risk patients (predicted probability > 0.708) had an actual postoperative complication rate of 78.2%, medium-risk patients (0.478–0.708) had a complication rate of 63.6%, and even the low-risk group had a rate of 28.8%. This stratification framework may serve as an exploratory auxiliary tool for preoperative risk communication and enhanced perioperative management but should not be used alone to determine whether to perform surgery; it must be combined with tumor burden, neurological compromise, expected survival, and patient preferences. Based on this, clinicians may consider the following interventions for high-risk patients: (1) implementation of immunonutritional support 7–14 days preoperatively; (2) development of prehabilitation exercise plans for low PMI patients; (3) enhanced postoperative monitoring, including early warning scores and low-threshold imaging examinations; (4) shared decision-making with patients and families, carefully weighing the benefits and risks of surgery versus non-surgical treatment. For medium-risk patients, enhanced perioperative monitoring and support are recommended; low-risk patients should also maintain routine monitoring without complete relaxation of vigilance. Although the above interventions have clinical rationality, their effectiveness in the lung cancer spinal metastases population still needs further verification in prospective studies.

This study has several limitations. Most importantly, our analysis did not fully integrate tumor biological characteristics. Although we described the distribution of histological subtypes, we were unable to include histological type in the regression model due to its imbalanced distribution. Moreover, key variables such as driver mutation status (e.g., EGFR, ALK, and KRAS), lines of prior systemic therapy, and primary disease control status were completely unavailable owing to the constraints of retrospective data collection. Therefore, our nomogram should be interpreted as a risk assessment tool based on host physiological reserve rather than a comprehensive model integrating tumor biological aggressiveness, and caution is warranted in clinical application. Second, as a single-center retrospective study with a limited sample size (n = 162) and no independent external validation cohort, the generalizability of the results requires confirmation in multicenter, large-sample studies. Third, a low PMI was defined solely based on muscle quantity measured by MRI, without incorporating muscle strength or physical performance; thus, it reflects low muscle mass rather than the full clinical syndrome of sarcopenia. Fourth, postoperative complications were only recorded up to 14 days after surgery, which may miss later events. Fifth, some potential confounders (e.g., specific chemotherapy regimens and socioeconomic status) were not included in the analysis.

## 5. Conclusions

In this cohort, low PMI, low BMI, low PNI, and low KPS showed potential associations with the occurrence of postoperative Clavien–Dindo ≥ grade II complications in patients with lung cancer spinal metastases. The nomogram demonstrated acceptable discrimination and calibration in internal validation; however, its predictive performance requires further confirmation through external validation. The exploratory risk stratification into three levels (low, medium, and high) based on tertiles of the model-predicted probability suggested a possible gradient of risk. As an exploratory analysis tool, this model may offer a preliminary quantitative reference for clinicians for the early identification of high-risk patients and the formulation of individualized perioperative management strategies, but its clinical utility requires confirmation in independent external cohorts.

## Figures and Tables

**Table 1:** Demographic and clinical baseline data of 162 patients with lung cancer spinal metastases.

Parameters	Statistics
Gender (*n*,%)	
Male	108 (66.7)
Female	54 (33.3)
DM or/and hypertension (*n*,%)	
Neither DM nor hypertension	93 (57.3)
Only hypertension	39 (24.1)
Only DM	15 (9.3)
Both DM and hypertension	15 (9.3)
Robotic assistance (*n*,%)	
Yes	69 (42.6)
No	93 (57.4)
Vertebral metastasis site (*n*,%)	
Cervical	30 (18.5)
Thoracic	75 (46.3)
Lumbar	57 (35.2)
Low PMI (*n*,%)	
Yes	118 (72.8)
No	44 (27.2)
Postoperative complications (*n*,%)	
<grade II	69 (42.6)
≥grade II	93 (57.4)
Tomita classification of tumor extent (*n*,%)	
Type 1	84 (51.9)
Type 2	9 (5.6)
Type 3	9 (5.6)
Type 4	33 (20.4)
Type 5	27 (16.5)
Histological subtype (*n*,%)	
Adenocarcinoma	123 (75.9)
Squamous cell carcinoma	21 (13.0)
Small cell lung cancer	18 (11.1)
Average age (years)	66.65 ± 9.85
BMI (kg/m^2^)	22.27 ± 3.76
PMI (cm^2^/m^2^)	3.12 ± 0.87
Preoperative PNI	44.53 ± 4.33
Preoperative KPS	55.19 ± 10.70
Preoperative HB level (g/L)	125.54 ± 14.28
Surgical duration (h)	2.76 ± 0.83
Instrumented levels	3.66 ± 1.27
Postoperative length of hospital stay (d)	「16 (7,25)」
Intraoperative blood loss (mL)	565.70 ± 140.77
Tokuhashi score	10.91 ± 1.90
Tomita score	4.62 ± 1.66

**Table 2:** Univariate analysis of binary logistic regression for postoperative complications.

Influencing Factors	Classification	β Value	Wald	*p* Value	OR Value	95% CI
DM or/and hypertension	Neither	0	4.867	0.182	1	
	Hypertension	−0.480	1.561	0.212	0.619	0.292–1.314
	DM	1.061	2.442	0.118	2.889	0.764–10.929
	Both	0.080	0.020	0.888	1.083	0.356–3.294
Low PMI		2.878	34.982	<0.001	17.774	6.849–46.126
BMI		−0.793	40.851	<0.001	0.452	0.355–0.577
Gender		0.229	0.454	0.501	1.257	0.646–2.447
Age		−0.019	1.358	0.244	0.981	0.950–1.013
Preoperative PNI		−0.900	40.923	<0.001	0.407	0.309–0.536
Preoperative KPS		−0.148	41.164	<0.001	0.862	0.824–0.902
Preoperative HB level		−0.031	6.991	0.065	0.969	0.822–1.116
Tokuhashi score		0.004	0.003	0.959	1.004	0.853–1.182
Instrumented levels		0.222	8.546	0.003	1.248	1.076–1.448
Surgical duration		0.264	7.788	0.005	1.302	1.082–1.567
Intraoperative blood loss (per 100 mL)		0.042	3.065	0.080	1.042	0.995–1.092
PMI		−0.178	0.924	0.337	0.837	0.582–1.204

Note: Reference group for comorbidities is “neither DM nor hypertension”; reference group for gender is “female”.

**Table 3:** The collinearity diagnosis of predictors.

Influencing Factors	Tolerance	VIF
Low PMI	0.958	1.044
BMI	0.621	1.610
Preoperative PNI	0.601	1.665
Preoperative KPS	0.641	1.559
Tokuhashi score	0.916	1.092
Instrumented levels	0.330	3.029
Surgical duration PMI	0.349 0.887	2.866 1.127
Intraoperative blood loss (per 100 mL)	0.384	2.601

**Table 5:** Association between stratification of independent risk factors and the incidence of postoperative Clavien–Dindo grade ≥ II complications.

Variable	Category	No. of Patients (*n*)	No. of Complications (*n*)	Complication Rate (%)	χ^2^ Value	*p* Value
PMI	High	44	6	13.636	47.332	<0.001
	Low	118	87	73.729		
PNI	High	81	18	22.222	82.022	<0.001
	Low	81	75	92.593		
BMI	High	81	21	25.926	65.663	<0.001
	Low	81	72	88.889		
KPS	High	82	29	35.366	32.993	<0.001
	Low	80	64	80.000		

Note: PNI stratification is based on the median (44.125) as the cutoff; BMI stratification is based on the median (21.772) as the cutoff; KPS stratification is based on the median (60.000) as the cutoff. All *p* values are from Pearson’s chi-square tests.

**Table 6:** Distribution of specific types of postoperative Clavien–Dindo ≥ grade II complications.

Complication Category	Number of Patients (*n*)	Proportion Among ≥ II Patients (%)
Infectious events	41	44.1
Hypoalbuminemia	59	63.4
Electrolyte disturbances	34	36.6
Thromboembolic events	16	17.2
Anemia	27	29.0
Others (pressure ulcer, arrhythmia, respiratory failure, etc.)	22	23.7

Note: Because some patients had multiple complications, the sum of percentages may exceed 100%.

**Table 7:** Postoperative complication rates by histological subtype.

Histological Subtype	No. of Patients (*n*)	No. of Complications (*n*)	Complication Rate (%)
Adenocarcinoma	123	69	56.1
Squamous cell carcinoma	21	12	57.1
Small cell lung cancer	18	12	66.7

Note: Descriptive analysis only. Due to small sample sizes in non-adenocarcinoma subgroups and lack of multivariable adjustment, direct statistical comparisons are not performed.

**Table 8:** Incidence of major complication types stratified by independent risk factors (%).

Complication Category	Low PNI (*n* = 81)	High PNI (*n* = 81)	Low PMI (*n* = 118)	High PMI (*n* = 44)
Infectious events	44.4	6.2	33.1	4.5
Hypoalbuminemia	67.9	4.9	47.5	6.8
Electrolyte disturbances	37.0	4.9	27.1	4.5
Thromboembolic events	17.2	2.5	12.7	2.3
Anemia	29.6	3.7	21.2	4.5
Others (pressure ulcer, arrhythmia, respiratory failure, etc.)	24.7	2.5	17.8	2.3

**Table 9:** Risk stratification by tertiles of nomogram-predicted probability and postoperative Clavien–Dindo grade ≥ II complications.

Risk Group	Predicted Probability Range	No. of Patients	No. of Complications	Complication Rate (%)
Low	<0.478	52	15	28.846
Medium	0.478–0.708	55	35	63.636
High	>0.708	55	43	78.182
Total	-	162	93	57.407

Note: chi-square test comparing complication rates among the three groups: χ^2^ = 27.929, df = 2, *p* < 0.001. After Bonferroni correction, pairwise differences in complication rates between any two groups were statistically significant (all *p* < 0.05).

## Data Availability

The data presented in this study are available on request from the corresponding author due to privacy restrictions.

## Abbreviations

The following abbreviations are used in this manuscript:

BMI	Body Mass Index
PNI	Prognostic Nutritional Index
KPS	Karnofsky Performance Status
PMI	Psoas Muscle Index
ALB	Albumin
TLC	Total Lymphocyte Count
HB	Hemoglobin
ROC	Receiver Operating Characteristic
AUC	Area Under the Curve
DCA	Decision Curve Analysis
H-L	Hosmer–Lemeshow
VIF	Variance Inflation Factor
ICC	Intraclass Correlation Coefficient
CI	Confidence Interval
OR	Odds Ratio
IQR	Interquartile Range
DM	Diabetes Mellitus
